# Aortopulmonary Fistula after Multiple Pulmonary Artery Stenting and Dilatation for Postarterial Switch Supravalvular Stenosis

**DOI:** 10.1155/2015/371925

**Published:** 2015-05-13

**Authors:** Maude Pagé, Oana Nastase, Frédéric Maes, Joëlle Kefer, Thierry Sluysmans, Alain Poncelet, Jean Rubay, Agnès Pasquet

**Affiliations:** ^1^Division of Cardiology, Cliniques Universitaires Saint-Luc, 1200 Brussels, Belgium; ^2^Division of Cardiology, Hôpital du Sacré-Coeur de Montréal, Montreal, QC, Canada H4J 1C5; ^3^Emergency Institute for Cardiovascular Diseases “Prof. C.C. Iliescu”, 022328 Bucharest, Romania; ^4^Division of Pediatric Cardiology, Cliniques Universitaires Saint-Luc, 1200 Brussels, Belgium; ^5^Division of Cardiac Surgery, Cliniques Universitaires Saint-Luc, 1200 Brussels, Belgium

## Abstract

We present a case of iatrogenic aortopulmonary fistula following pulmonary artery (PA) stenting late after arterial switch operation (ASO) for D-transposition of the great arteries (D-TGA), an unusual complication that may be encountered more frequently in contemporary adult cardiology clinics. The diagnosis should be sought in the face of unexplained heart failure in patients who underwent ASO and subsequent PA angioplasty. Treatment should be instituted in a timely fashion, and options include surgical correction or implantation of a duct occluder or covered stent.

## 1. Case Report

A 27-year-old patient with surgically corrected D-TGA was admitted with dyspnea, ascites, and pleural effusions. He had benefited from an arterial switch operation (ASO) with Lecompte manoeuvre in the neonatal period, with transannular patch enlargement of main pulmonary artery (PA) one year later.

At age 7, he underwent balloon angioplasty and stenting (*Palmaz*, Johnson & Johnson-Cordis, Bridgewater, NJ, USA) of the right pulmonary artery (RPA), and this stent was later redilated, concomitantly with left PA angioplasty and stent implantation (*mega-LD*, ev3, Plymouth, Minnesota) mounted on a 14 mm balloon-in-balloon catheter. At age 24, stenoses at the origins of both PAs were addressed with high-pressure inflations and stenting (*Maxi LD,* ev3, Plymouth, Minnesota), mounted on a 16 mm balloon-in-balloon catheter, of the main PA. One year later, the patient complained of fatigue, and exercise testing objectivised decreased maximal oxygen consumption (18.5 mL/kg/min; 40% predicted). Due to recurrent stenosis of the main PA as assessed at transthoracic echocardiography (TTE), a kissing balloon dilatation was performed using an* Atlas* 16 mm in the LPA and 14 mm in the RPA, without any complication reported.

**Figure 1:**
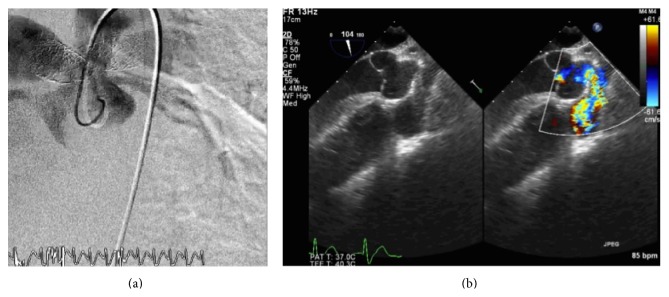
Angiogram with contrast injection in the ascending aorta showing simultaneous opacification of the RPA (a); mid oesophageal long axis showing a communication between the ascending aorta and the RPA (b).

There was then a 16-month interval before the current admission for heart failure. After a TTE assessment, cardiac magnetic resonance (CMR) confirmed right ventricular (RV) dilatation (end-diastolic volume (EDV) index 193 mL/m^2^, ejection fraction (EF) 46%) and new left ventricular (LV) dilatation (EDV index 191 mL/m^2^, EF 52%) with backward flow (77 mL) in the ascending aorta at phase contrast velocity mapping, which was discordant with the absence of aortic regurgitation at TTE. CMR was also remarkable for significant pulmonary regurgitation and did not show any delayed gadolinium enhancement suggestive of myocardial fibrosis. Because of apparently increased gradient across the pulmonary outflow at TTE, a decision to take the patient to the catheterization laboratory was made. Aortography was remarkable for simultaneous opacification of the PAs (Figure 1(a), video 1 in the Supplementary Material available online at http://dx.doi.org/10.1155/2015/371925). A* Judkin* right catheter and* Terumo* wire allowed the demonstration of a fistula between the ascending aorta and the RPA stent, establishing the diagnosis of aortopulmonary window, confirmed by oximetric step-up (RV SpO2 77.6% versus RPA SpO2 92.9%; Qp:Qs 4.9). Transesophageal echocardiogram (TEE) better defined the fistulous path (Figure 1(b)).

**Figure 2:**
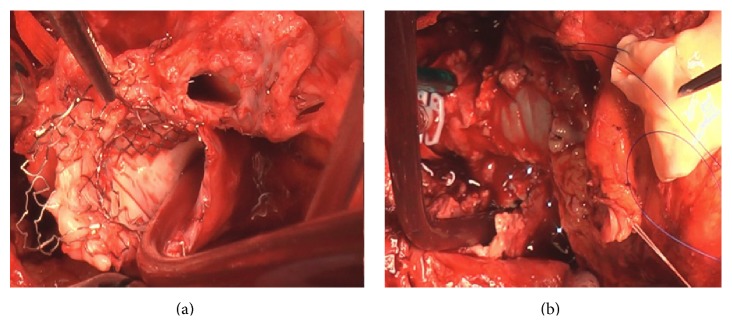
Large aortopulmonary fistula originating from the proximal part of the RPA stent (a); patch closure of the defect (b).

Due to the presence of both coronary ostia in the vicinity of the fistula, the patient was scheduled for a surgical correction. After resternotomy, cardiopulmonary bypass was initiated using right subclavian artery and bicaval cannulation. Mild hypothermia and retrograde cardioplegia through the coronary sinus were used. The brachiocephalic vein, superior vena cava, distal ascending aorta, and PAs were dissected. After cross clamping and cardioplegic arrest, the distal main PA and the transannular patch were incised. A 1 cm defect in the RPA communicating with the anterior aspect of the ascending aorta, adjacent to the right coronary ostium, was found (Figure 2(a)). The defect in the aortic wall was repaired with bovine pericardium (St. Jude Medical). Bilateral stents extraction and reconstruction of the RV to PAs continuity  were performed using a bifurcated pulmonary valve homograft conduit (Figure 2(b)). Weaning from CPB required peripheral extracorporeal membrane oxygenation due to severe RV dysfunction, which was withdrawn at postoperative day 7. Follow-up TTE revealed partial RV recovery, normal homograft function, and laminar PA flows. He remained hemodynamically stable thereafter.

## 2. Discussion

This case illustrates a potentially underrecognized complication of PA stenting after ASO, which has replaced the Senning and Mustard procedures for treatment of D-TGA, allowing reestablishment of ventriculoarterial concordance. The Lecompte manoeuvre involves moving the PA bifurcation anterior to the aorta. Despite improved long-term outcomes compared to atrial switch, supravalvular pulmonary stenosis occurs in 17–55% of patients, although recent advances in the operative technique improved the midterm outcomes [1, 2]. Nevertheless, this complication is most commonly treated with balloon dilatation and stenting of the PAs.

In our patient, repeated stenting and dilatation in the RPA led to the creation of a fistula and left-to-right shunt with chamber dilatation. At review of the TTEs, a color flow between the aorta and PA may be suspected, which had been misdiagnosed as flow acceleration in the context of PA stenosis and pulmonary regurgitation (video 2). LV dilatation at CMR and backward flow in the axis of the ascending aorta without aortic regurgitation at TTE were suggestive of the diagnosis, which was definitively established at catheterization.

Very few cases of aortopulmonary window following PA stenting after the ASO are reported. Chiostri et al. reported the case of a 3-year-old boy in whom balloon dilatation of a postoperative pulmonary stenosis disclosed an unsuspected but previously present aortopulmonary fistula [3]. In this case, surgical treatment was performed due to failure at closing the fistula in the catheterization laboratory by duct occluder. Iatrogenic aortopulmonary fistula after ASO and transcatheter stent placement for a left PA stenosis has also been reported in an adolescent in whom postprocedural symptoms were first attributed to pulmonary reperfusion oedema and the right diagnosis established four months later due to ongoing symptoms. A covered stent successfully occluded the defect and the patient had a favourable outcome [4]. Such a complication has also been reported in a 10-year-old boy who had developed a left PA stenosis 9 years after ASO, which was treated by balloon angioplasty, initially successful although the patient had suffered unilateral pulmonary oedema and a rise in PA pressure at the end of the procedure. The diagnosis of aortopulmonary window was made at multimodality imaging involving echocardiography, CMR, and cardiac catheterization one year later when the patient presented with signs and symptoms of congestive heart failure. A covered stent then successfully treated the fistula and the residual left PA stenosis [5].

Given the relatively high incidence of supravalvular pulmonary stenosis after ASO and the increasing survival into adulthood of this population, iatrogenic aortopulmonary fistulae may be encountered more frequently in contemporary adult cardiology practice. Accordingly, a high level of suspicion is advised in the context of unexplained LV or RV failure, and appropriate treatment must be instituted in a timely fashion to avoid irreversible damage of large left-to-right shunting.

## Supplementary Material

Video 1. Aortography with contrast injection in the ascending aorta showing simultaneous opacification of the aorta and the right pulmonary artery, establishing the diagnosis of aortopulmonary window.
Video 2. Trans-thoracic short axis view, performed 16 months prior to the current admission, showing flow aliasing in the axis of the main and right pulmonary artery.
